# Physicochemical Properties and Bacterial Community Profiling of Optimal *Mahewu* (A Fermented Food Product) Prepared Using White and Yellow Maize with Different Inocula

**DOI:** 10.3390/foods11203171

**Published:** 2022-10-11

**Authors:** Grace Abosede Daji, Ezekiel Green, Adrian Abrahams, Ajibola Bamikole Oyedeji, Kedibone Masenya, Kulsum Kondiah, Oluwafemi Ayodeji Adebo

**Affiliations:** 1 Food Innovation Research Group, Department of Biotechnology and Food Technology, Faculty of Science, University of Johannesburg, Johannesburg 2028, South Africa; 2 Department of Biotechnology and Food Technology, Faculty of Science, University of Johannesburg, Johannesburg 2028, South Africa; 3 Neuroscience Institute, University of Cape Town, Private Bag X3, Rondebosch, Cape Town 7701, South Africa

**Keywords:** fermentation, optimisation, response surface methodology, metagenomics, *mahewu*, maize

## Abstract

*Mahewu* is a fermented food product from maize, commonly consumed in Southern Africa. This study investigated the effect of optimizing fermentation (time and temperature) and boiling time of white maize (WM) and yellow maize (YM) *mahewu*, with the use of the Box–Behnken-response surface methodology (RSM). Fermentation time and temperature as well as boiling time were optimized and pH, total titratable acidity (TTA) and total soluble solids (TSS) determined. Results obtained showed that the processing conditions significantly (*p* ≤ 0.05) influenced the physicochemical properties. pH values of the *mahewu* samples ranged between 3.48–5.28 and 3.50–4.20 for YM *mahewu* and WM *mahewu* samples, respectively. Reduction in pH values after fermentation coincided with an increase in TTA as well as changes in the TSS values. Using the numerical multi-response optimisation of three investigated responses the optimal fermentation conditions were observed to be 25 °C for 54 h and a boiling time of 19 min for white maize *mahewu* and 29 °C for 72 h and a boiling time of 13 min for yellow maize *mahewu*. Thereafter white and yellow maize *mahewu* were prepared with the optimized conditions using different inocula (sorghum malt flour, wheat flour, millet malt flour or maize malt flour) and the pH, TTA and TSS of the derived *mahewu* samples determined. Additionally, amplicon sequencing of the 16S rRNA gene was used to characterise the relative abundance of bacterial genera in optimized *mahewu* samples, malted grains as well as flour samples. Major bacterial genera observed in the *mahewu* samples included *Paenibacillus*, *Stenotrophomonas*, *Weissella*, *Pseudomonas*, *Lactococcus*, *Enterococcus, Lactobacillus, Bacillus*, *Massilia*, *Clostridium sensu stricto 1, Streptococcus*, *Staphylococcus*, *Sanguibacter*, *Roseococcus*, *Leuconostoc*, *Cutibacterium*, *Brevibacterium*, *Blastococcus*, *Sphingomonas* and *Pediococcus*, with variations noted for YM *mahewu* and WM *mahewu*. As a result, the variations in physicochemical properties are due to differences in maize type and modification in processing conditions. This study also discovered the existence of variety of bacterial that can be isolated for controlled fermentation of *mahewu*.

## 1. Introduction

Maize is one of the topmost cereal crops identified as a major source of food in many countries [1]. Maize is transformed into different food products using fermentation and such fermented foods including *kenkey*, *kwete, munkoyo, uji* and *ogi*, form part of the culture and nutrient sources of inhabitants of under-developed countries [2]. Fermented foods include beverages produced through microbial growth and the conversion of food components through enzymatic action. These beverages often possess considerably high nutritive value, pleasant texture, shelf life, aroma, flavour, and health-promoting components which makes them highly appealing to consumers [3].

These fermented beverages are widely consumed, contributing significantly to the safety and diversity of the human diet, particularly in Africa and Asia [4]. The best adapted strain from a mix of microorganisms (bacteria, yeast and fungi) determines the safety, stability and overall quality in several fermented foods [5,6]. However, probiotic microorganisms have been isolated from fermented foods [7]. The majority of such probiotic microorganisms belong to lactic acid bacteria and bifidobacteria [7,8]. Interestingly, traditionally fermented African cereal-based beverages are potential probiotic carriers, due to the presence of some probiotic strains such as *Lactobacillus* spp. which are involved in the fermentation of such products. This offers an opportunity for the African cereal beverages to be used to provide probiotic health benefits to the majority of African populations [9].

*Mahewu,* a fermented maize-based product is an important indigenous fermented maize product predominantly consumed in South Africa and other neighbouring countries. *Mahewu* is generally a cream-white fermented drink when made from white maize or could be pale yellow when made from yellow maize, due to the inherent colour of the maize. Both white and yellow maize derived *mahewu* were investigated in this study. It is a highly refreshing and hunger-filling drink used as weaning foods for infants as well as in social functions in some African countries [2]. As seen in the literature, there are several kinds of research on the fermentation and boiling conditions reported for the preparation of *mahewu* [10,11,12] and these different conditions are yet to be optimised or standardised. This has led to variations in the quality and stability of the product.

The response surface methodology (RSM) is an assembly of mathematical and statistical procedures, which is used to generate various optimisation conditions to achieve reproducible results [13]. The Box–Behnken design (BBD) of RSM does not only explore the whole of an experimental domain but is also easily applied to optimise variables more effectively for better and stable quality of *mahewu* product [14].

The microbial profile of *mahewu* has been mostly determined using culture-dependent approaches, such microbes were identified as *Lactobacillus brevis*, *Lactobacillus casei*, *Lactobacillus plantarum*, *Lactococcus lactis*, *Leuconostoc lactis* and *Pediococcus pentosaceus* [12,15]. Again, it appears that these approaches did not explore the optimisation of fermentation and boiling variables. These scientists underlined the need for technological improvement within the area of food safety and processing to enhance the potential of this technology [16,17,18]. Therefore, whole genome deoxyribonucleic acid (DNA) extraction from fermented foods and subsequent determination of their microbiota, usually referred to as metagenomics, is a powerful technology for the identification and characterisation of the ecosystems of both dominant and less prevalent genera of microorganisms and can be useful in the determination of the bacterial community of fermenting organisms in *mahewu* [14]. This study was thus carried out to optimise the fermentation (time and temperature) and boiling time of *mahewu* from white and yellow maize and to evaluate their bacteria composition.

## 2. Materials and Methods

### 2.1. Raw Materials and Sample Preparation

White maize (WM) (*Zea mays* L.) was purchased from Driefontein in Mpumalanga province of South Africa, yellow maize (YM) was purchased from Drover investment (Pty) Ltd., Newton in Gauteng province of South Africa, sorghum (*Sorghum bicolor* L.), wheat (*Triticum*) and finger millet (*Eleusine coracana*) grains were purchased from Kingco Pty (Ltd), Johannesburg, South Africa.

Malted grains were thoroughly washed with distilled water and steeped for 24 h (sorghum and millet) and 48 h (white and yellow maize) at room temperature. Water was drained off and the soaked seeds were germinated at 25 °C for 24 h for MS (malted sorghum) and MM (millet malt) and 72 h for MWM (malted white maize) and MYM (malted yellow maize), in an incubator (small baking oven-K. Huber Engineering, New Delhi, India). Sprouted seeds were evenly spread on a tray in an air oven (Prolab Hot Air Oven, New Delhi, India,) and dried at 60 °C for 24 h. The sprouts were removed manually and winnowed. The seeds were then milled (Perten Laboratory Mill 3310 Instruments AB, Helsinki, Finland) into fine flour and packaged into zip lock bags for further use. Unmalted white, yellow and wheat grains were sorted, cleaned, milled and packaged into Ziplock bags for further use.

### 2.2. Processing of Maize into Mahewu

*Mahewu* was prepared using the modified method of Awobusuyi et al. (2016) [11]. The maize flour (70 g) (WM or YM) was boiled in water (7:100 *w*/*v*) at 90 °C into porridge at times specified in Table 1, with occasional stirring. The resulting porridge was cooled to 25 ± 2 °C and 5% of inoculum (malted sorghum) was added and allowed to ferment at the time and temperature conditions described in Table 1. Each sample was subjected to pH, total titratable acidity (TTA) and total soluble solids (TSS) analyses.

### 2.3. The Optimisation of Parameters to Produce Mahewu

Different parameters such as boiling time (*X*_1_), fermentation temperature (*X*_2_) and fermentation time (*X*_3_), with ranges of 10–20 min, 25–45 °C and of 16–72 h, respectively, were investigated. The choice of the parameters investigated was based on previous studies on the production of *mahewu* [12,15,16]. The three-factor Box–Behnken design gave a total of 17 experimental runs each for white and yellow maize, respectively (Table 1). Three responses, including pH (*Y*_1_), TTA (*Y*_2_) and TSS (*Y*_3_) were investigated.

The Box–Behnken general second-order polynomial model in Equation (1) was used to depict the mathematical expression of the relationship between the process variables, including linear, quadratic and interactive effects.

(1) $$Y=\beta_{0}+\beta_{1}X_{1}+\beta_{2}X_{2}+\beta_{3}X_{3}+\beta_{11}X_{1}^{2}+\beta_{22}X_{2}^{2}+\;\beta_{33}X_{3}^{2}+\;\beta_{12}X_{1}X_{2}+\beta_{13}X_{1}X_{3}+\beta_{23}X_{2}X_{3}$$

where *Y* is the response, *X*_1_, *X*_2_, *X*_3_ are factors, *β*_0_…*β*_33_ are the coefficients of linear, quadratic and interaction terms. The response surfaces were represented with model equations and respective coefficients obtained using the Box–Behnken-response surface methodology. 

### 2.4. pH, TTA and TSS

The pH of each *mahewu* sample was measured after the fermentation process using a pH meter (Hand-Held EcoSense pH10An pen Tester, Beijing, China). The titratable acidity (TTA) analysis was carried out by titrating 2 g of *mahewu* sample mixed in 20 mL distilled water against 0.1 N NaOH, with the use of phenolphthalein as an indicator, the result obtained was expressed in percentage (% lactic acid). The total soluble solids (TSS) determination was carried out using a refractometer (Hanna H196801, Woonsocket, RI, USA), and the results were expressed as °Brix.

### 2.5. Multi-Response Numerical Optimisation and Processing of Optimal Mahewu Samples

Based on the experimental results obtained from Table 1, a numerical multi-response optimisation of parameters (pH, TTA, TSS) was carried out on Minitab 16 (Minitab Lt. Coventry, UK). The optimal processing conditions obtained were a fermentation temperature of 25 °C for 54 h and a boiling time of 19 min for white maize *mahewu* while that of yellow maize *mahewu* were fermentation conditions of 29 °C for 72 h and a boiling time of 13 min. For both white and yellow maize, a total of eight (8) optimised *mahewu* samples with different maize grains (white and yellow) and various inocula were obtained; (i) WM *mahewu* with MS, (ii) WM *mahewu* with wheat, (iii) WM *mahewu* with MM, (iv) WM *mahewu* with MWM, (v) YM *mahewu* with MS, (vi) YM *mahewu* with wheat, (vii) YM *mahewu* with MM, (viii) YM *mahewu* with MYM.

### 2.6. Investigation of Parameters in Optimized Mahewu Samples

#### 2.6.1. pH, TTA and TSS 

The pH, TTA and TSS were analysed as described earlier in Section 2.4. Thereafter, samples were freeze-dried at −55°C under a vacuum (Telstar LyoQuest, Labotec, Midrand, South Africa) for 24 h.

#### 2.6.2. Profiling of the Bacterial Community

##### The ZymoBIOMICS^TM^ DNA Extraction

The bacterial DNA extraction was performed using a ZymoBIOMICS^TM^ DNA miniprep kit (Inqaba Biotech, Pretoria, South Africa) according to the manufacturer’s instructions. DNA samples from raw maize flours, optimal *mahewu* samples and inocula used were extracted. The extracted DNA was stored at −80 °C for further processing. Subsequently, the quantity and quality of the eluted DNA was verified using nano-drop equipment (Implen Nano-photometer N60—Touch, Cape Town, South Africa). The extracted DNA was subsequently sent to Inqaba Biotech Pty (Ltd), Johannesburg, South Africa, for 16s rRNA sequencing.

##### 16s rRNA Amplicon Sequencing

The total DNA extracted earlier from samples was amplified at the 16S region using universal primers 5ʹ-CCTACGGGNGGCWGCAG-3ʹ and 5ʹ-GACTACHVGGGTATCTAATCC-3. The hypervariable regions targeted were V3-V4. The reaction was carried out in 50 µL volumes containing 0.3 mg/mL BSA (Bovine Serum Albumin), 250 µM dTNPs, 0.5 µM of each primer, 0.02 U Phusion High-Fidelity DNA Polymerase (Finnzymes OY, Espoo, Finland) and 5× Phusion HF buffer containing 1.5 mM MgCl_2_ on a GeneAmp PCR system using the following PCR conditions: initial denaturation at 95 °C for 5 min, 25 cycles of denaturation at 95 °C for 40 s followed by annealing for 2 min, extension at 72 °C for 1 min and a final step of extension at 72 °C for 7 min. Subsequently, PCR products were purified in an ExoSAP, Affymetrix, Inc. Santa Clara, CA, USA. The amplicon library was normalised and prepared for sequencing following the 16S rRNA gene library preparation guide (Amplicon, 2013). Sequencing was performed using an Illumina MiSeq Sequencer (Illumina, San Diego, CA, USA) with a MiSeq Reagent Kit v3 to generate 2 × 300 paired end reads at Inqaba Biotech Pty (Ltd), Johannesburg, South Africa.

##### Bioinformatics Analysis

The 16S sequencing data included the forward and reverse paired-end reads. The demultiplexed forward and reverse reads were imported with the CASAVA 1.8 pipeline (paired-end) using the Quantitative Insights into Microbial Ecology package (Qiime2) The demux.qzv visualisation provided the length of nucleotides to trim and truncate for the subsequent qiime denoise analysis. This further displayed the data quality scores, allowing the removal of low-quality reads <Phred33 with the deblur plugin. Sequence variant calling of the illumina-amplicon sequences and chimeric sequences was removed using deblur plugin.

The compositional and taxonomic analyses were conducted by using feature-classifier plugins, i.e., composition and taxa (https://github.com/qiime2/q2) (accessed on 24 May 2021) using a pretrained Naive-Bayes classifier SILVA 138. Subsequently, sequences were clustered according to similarities into an operational taxonomic unit (OTU), followed by the generation of a representative sequence for each OTU. Taxonomy of the resulting OTU was used for downstream taxonomic assignment. A phylogenetic tree with the phylogeny fasttree command was generated with the use of the feature table. Graphical representations were conducted using the phyloseq package with the Bioconductor version 3.0 and R version 3.5.1.

### 2.7. Statistical Analysis

Except for the profiling of the microbial community done with R software (R-4.1.2 for Windows), all other analyses were done in triplicate using the analysis of variance (ANOVA) in the Minitab 16 software (Minitab Lt. Coventry, UK) and differences were considered statistically significant if *p* ≤ 0.05.

## 3. Results

### 3.1. pH, TTA and TSS

The role and importance of maize-based diets in the provision of health benefits is key to achieving food security, hence, the selection of a suitable model to generate the optimum conditions of factors for desirable *mahewu* products is necessary. Experimental results of the seventeen runs generated by the BBD (Table 2) for each maize-based product (YM and WM) revealed that pH values decreased with an equivalent increase in TTA.

The pH values of the maize-based products (YM and WM) ranges from 3.48 to 5.28 for YM and 3.50 to 4.20 for WM (Table 2). It was observed that *mahewu* samples prepared for longer times (44–72 h) at 25 and 35 °C had lower pH values compared to those with the shorter fermentation time (16 h). It is also expected that at a low pH value, the growth of most spoilage bacteria can be inhibited [19]. Besides, at 45 °C the highest pH values were recorded for both maize types. As expected in all samples, as the pH values decreased, there was a corresponding increase in TTA, which is indicative of the acid fermentation that is expected to occur. Additionally, at the end of fermentation, the TSS values decreased from approximately 5.90 °Brix to 4.43 °Brix for white maize *mahewu* and 5.87 °Brix to 4.10 °Brix for yellow maize *mahewu*.

The differences in pH, TTA and TSS values for the experimental runs of the maize-based products (WM and YM *mahewu*) could be attributed to differences in cooking time, fermentation conditions as well as sample source and inoculum. It is important to note that in fermented beverages, pH, TTA and TSS are significant in determining microbial stability against food-borne pathogens and correlate with the taste of the products. Subsequently, the seventeen experimental results of the different types of *mahewu* products (Table 2) were used to generate an optimal condition for both WM and YM for the preparation of *mahewu* as described in Section 2.5.

### 3.2. Statistical Models and Validation

This study investigated the effects of independent process variables (fermentation temperature (*X*_1_), fermentation time (*X*_2_) and boiling time (*X*_3_)) of WM and YM on the production of *mahewu*. For numerical optimisation, the parameters determined were pH (*Y*_1_), TTA (*Y*_2_) and TSS (*Y*_3_) and the Box–Behnken model equations representing each are provided in Equations (2)–(4) for white maize and Equations (5)–(7) for yellow maize.

(2) $$Y_{1}=5.70414-0.06275X_{1}-0.02664X_{2}-0.08255X_{3}+0.00113X_{1}^{2}+0.00019X_{2}^{2}+0.00321X_{3}^{2}\;+0.00016X_{1}X_{2}-0.00020X_{1}X_{3}-0.00013X_{2}X_{3}$$

(3) $$Y_{2}=-0.950000+0.043339X_{1}+0.012366X_{2}+0.063821X_{3}-0.000725X_{1}^{2}-0.000089X_{2}^{2}-0.002300X_{3}^{2}\;-0.000045X_{1}X_{2}+0.000000X_{1}X_{3}+0.000089X_{2}X_{3}$$

(4) $$Y_{3}=3.56018+0.02193X_{1}+0.00673X_{2}+0.00871X_{3}-0.00111X_{1}^{2}+0.00018X_{2}^{2}-0.00165X_{3}^{2}\;+0.00004X_{1}X_{2}+0.00485X_{1}X_{3}-0.00161X_{2}X_{3}$$

(5) $$Y_{1}=7.47591-0.30732X_{1}+0.01592X_{2}+0.04174X_{3}+0.00542X_{1}^{2}+0.00003X_{2}^{2}-0.00074X_{3}^{2}\;-0.00037X_{1}X_{2}+0.0004X_{1}X_{3}-0.00077X_{2}X_{3}\;$$

(6) $$Y_{2}=-1.12260+0.10172X_{1}+0.00988X_{2}-0.03239X_{3}-0.00146X_{1}^{2}-0.00004X_{2}^{2}+0.00107X_{3}^{2}\;-0.00022X_{1}X_{2}-0.00010X_{1}X_{3}+0.00014X_{2}X_{3}\;$$

(7) $$Y_{3}=3.90414-0.01424X_{1}+0.04825X_{2}-0.03851X_{3}+\;0.00126X_{1}^{2}-0.00035X_{2}^{2}\;+0.00781X_{3}^{2}-0.00006X_{1}X_{2}-0.00365X_{1}X_{3}-0.00095X_{2}X_{3}\;\;$$

All calculated R^2^ values in this study were above 80% (Table 3) which signifies a good model fit as reported in the literature [13,20]. Lower R^2^ values in this study could be explained by the reduction in TSS after fermentation, which could be due to the loss of nutrients during the germination process of the inoculum (sorghum malt). This makes sense because sprouting, as a metabolic process, involves nutrient breakdown and is also accompanied with the growth of cotyledons and the release of energy [21]. This finding is in line with the findings in the literature [21].

To obtain the optimum conditions in each case, the degree of importance of each of the responses (pH, TTA and TSS) were defined in the Minitab software to obtain the desired result in the resulting *mahewu* samples. Hence, the TSS was minimised while targeted values of 3.5 for pH and 0.5% for TTA were defined. The optimal processing conditions obtained were a fermentation temperature of 25 °C for 54 h and a boiling time of 19 min for white maize *mahewu* with desirability factor (D_f_) of 0.95, while the fermentation conditions of 29 °C for 72 h and a boiling time of 13 min with desirability factor (D_f_) of 0.86 were obtained for yellow maize *mahewu*.

These optimal conditions were further used to investigate the pH, TTA and TSS on the eight different types of *mahewu* samples produced. Experimental results of the eight samples generated for each maize-based product revealed that pH values decreased (increased acidity) with an equivalent increase in TTA (Table 4). The inverse proportional trend between pH and titratable acidity has been previously reported by several scientists [13,22].

Therefore, these results suggest an accumulation of organic acids with an increase in the activity of microbes and metabolism of the fermenting organisms. The pH of the optimal maize-based products (WM and YM *mahewu*) ranged between 3.41 to 4.51 for WM (Table 4) and 3.44 to 4.65 for YM (Table 4), whereas their TTA ranged between 0.47 to 0.68% for WM and 0.38 to 0.62% for YM. With significant differences (*p* ≤ 0.05) in the values obtained. These differences could be due to their difference in fermentation time and temperature which made the *mahewu* samples ferment differently. Interestingly, the values reported herein are within the acceptable range reported for *mahewu* and other fermented cereal beverages [11,15,23,24,25].

The TSS is an approximate measurement of sugar content. Boiling the *mahewu* for an adequate measure of time is essential for starch gelatinisation and release of locked-up nutrients in yeast cells [26]. Therefore, the proliferation of fermentative microbes is driven by the hydrolysis of cooked starch to fermentable sugars by endogenous amylolytic enzymes [25]. As observed, the general pattern for all *mahewu* samples herein (Table 4), showed that TSS decreased from approximately 5.90 °Brix to the experimental values obtained for both white and yellow maize *mahewu* at the end of the fermentation process. A similar trend has been documented [24,27]. This decrease in TSS at the end of fermentation could be because of the high bacterial load in the *mahewu* samples, which meant rapid utilisation of accessible solids [28]. Furthermore, fermentation of sugars might have caused lactic acid bacteria to multiply as acidity increased [29].

### 3.3. Profiling of Bacterial Community in Optimized Mahewu Samples

**Figure 1:**
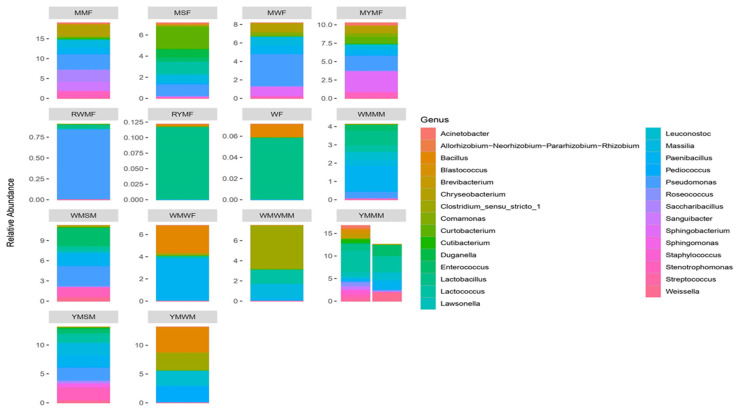
Bacterial taxa at the genus level for optimised *mahewu* samples. MMF—malted millet flour, MSF—malted sorghum flour, MWF—Malted white maize flour, MYMF—malted yellow maize flour, RWMF—raw white maize flour, RYMF—raw yellow maize flour, WF—wheat flour, WMMM -white maize *mahewu* with malted millet, WMSM—white maize *mahewu* with sorghum malt, WMWF—white maize *mahewu* with wheat flour, WMWMM—white maize *mahewu* with white maize malt, YMMM—yellow maize *mahewu* with malted millet, YMSM—yellow maize mahewu with sorghum malt, YMW—yellow maize mahewu with wheat.

16S amplicon analysis showed the first report on the bacterial community present in optimised *mahewu* samples (Figure 1) and they included *Paenibacillus*, *Stenotrophomonas*, *Weissella*, *Pseudomonas*, *Lactococcus*, *Enterococcus, Lactobacillus, Bacillus*, *Massilia*, *Clostridium sensu stricto 1, Streptococcus*, *Staphylococcus*, *Sanguibacter*, *Roseococcus*, *Leuconostoc*, *Cutibacterium*, *Brevibacterium*, *Blastococcus*, *Sphingomonas* and *Pediococcus*. Nevertheless, the difference in bacterial composition between yellow maize *mahewu* and white maize *mahewu* could be due to the differences in cooking time, fermentation conditions as well as sample source and inoculum. However, bacterial composition was low in raw white maize flour (RWMF), raw yellow maize flour (RYMF) and wheat flour (WF).

Few studies have reported members of the genera *Weissella*, *Leuconostoc, Lactobacillus*, *Streptococcus, Enterococcus*, *Lactococcus* and *Pediococcus* as dominant lactic acid bacteria in fermented foods [30,31,32,33]. This was expected since all products exhibited a pH of around 3.5 to 4.6. *Weissella* and *Leuconostoc* species are heterofermenters that produce ethanol and CO_2_ in addition to organic acids [33]. *Lactobacillus* and *Streptococcus* species are homofermenters producing lactic acid as a major product [33]. Members of the *Leuconostoc* genera have been considered as a starter to produce commercially fermented *kimchi* with good quality. It was reported that *Enterococcus* is active in the natural fermentation of *meju*, indicating that the bacteria may exist extensively in some fermentation steps of *meju* [30]. In another study, *Lactococcus* and *Pediococcus* were reported to be present in *mahewu*. Consistent with other studies, the bacterial community detected in this study has been previously reported in fermented products [10,34,35,36,37,38,39,40,41].

The presence of *Clostridium sensu stricto 1* enhances the production of butyrate, which induces the production of regulatory T cells and play an anti-inflammatory role [42]. Additionally, Tao and his colleagues reported that during fermentation, *Clostridium sensu stricto 1* stimulated the proliferation of fibrolytic bacteria, which in turn degraded fibres to produce organic acid and monosaccharides [43]. Interestingly, it has been reported that the presence of lactic acid bacteria in fermented food may drastically reduce the duration and severe effects of gastrointestinal disorders [44]. It has also been established that some LAB-fermented foods have antimutagenic and anticarcinogenic activities [45,46,47].

### 3.4. Profiling of Bacterial Community in Malt and Flour Samples

**Figure 2:**
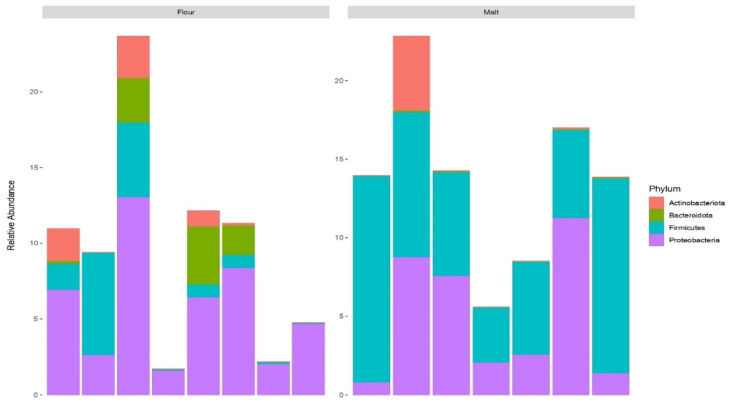
Bacterial taxa at the phylum level for flour and malt samples.

**Figure 3:**
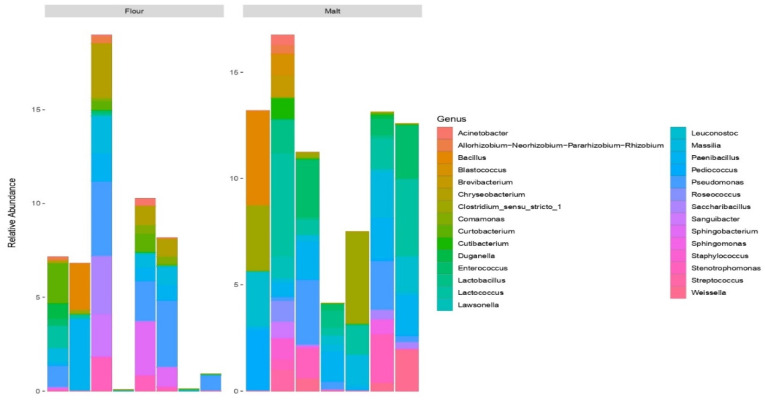
Bacterial taxa at the genus level for flour and malt samples.

To determine their relative abundance, samples were characterised by the analysis of different bacterial taxa at the phylum and genus level. *Proteobacteria* was the most abundant and prevalent phylum in the flour sample whereas *Firmicutes* was most prevalent in the malt samples followed by *Proteobacteria* (Figure 2) with *Pseudomonas* as the most abundant genus in the flour samples while *Lactococcus* was the most prevalent in the malt samples followed by *Paenibacillus* (Figure 3). The members of the phylum Proteobacteria and genus *Pseudomonas* have been reported in wholemeal wheat flours and barley grain [46,48]. Members of the phyla Firmicutes and Proteobacteria, as well as the genera *Lactococcus* and *Paenibacillus* found in malt samples in this study, have previously been reported in the literature [49,50]. The taxonomic classification of the lactic acid bacteria such as *Weissella*, *Streptococcus*, *Pediococcus*, *Leuconostoc*, *Lactococcus*, *Lactobacillus* and *Enterococcus* as revealed herein were the most dominant in the malt samples. In the same line, the interactions of lactic acid bacteria in food are typically involved in spontaneous fermentation and plays key role in food safety.

Nevertheless, the significant decrease and increase in pH and TTA, respectively, after the fermentation of *mahewu* prepared with the malt samples as compared to *mahewu* prepared with wheat flour could be because of the hydrolysis of some complex organic molecules such as phytin, protein and lipids to acetic acid, lactic acid, fatty acids, phosphate and amino acids during malting and fermentation. Similar observations have been reported in the literature by Muhammed and his co-workers on their work on wheat and millet [51].

Hence, the decrease in pH and increase in acidity accompanying fermentation could be attributed to the extent of these complex molecules as well as the digestibility of the malted and fermented wheat and millet. This is consistent with the work documented by Muhammed and his co-workers on their work on maize [51]. Additionally, the higher bacteria load in this samples led to reduced TSS values.

Bacterial composition was low in raw white maize flour (RWMF), raw yellow maize flour (RYMF) and wheat flour (WF) but a higher bacterial composition in the malted inocula may be the result of the malting process. With respect to microbial activity and safety, steeping is the most critical step in malting [52]. Microbes, particularly lactic acid bacteria rapidly increase due to the limited accessibility of oxygen during steeping [53]. During germination, the ability of bacteria constituents to form a complex matrix in the form of a biofilm make the bacteria taxa a dominant microflora. Additionally, bacteria constituents have been reported to be the dominant microflora during germination [54]. Additionally, metabolic changes which include the conversion of residual carbohydrates to fermentable sugars occur during germination. In this phase, *Lactobacilli* was found as the predominant LAB, whereas *Leuconostoc* species were the major LAB during steeping. Furthermore, kilning which happens to be the final phase during malting is very important to the quality and stability of microbes [54]. However, the microbes that present at this phase are certain heat resistant bacteria which can form spores and they include *bacillus* species [51]. Similarly, as reported in the literature, malting can significantly improve the grain nutritional profile such as proteins, phenolic compounds as well as functional microorganisms with health benefits [55].

## 4. Conclusions

It could be concluded that the optimum processing conditions for white and yellow maize *mahewu* are 25 °C for 54 h with a boiling time of 19 min for white maize *mahewu* and 29 °C for 72 h with a boiling time of 13 min for yellow maize *mahewu*. Isolation of bacterial nucleic acids from the natural environment has become a useful tool to detect bacteria that may be difficult to culture. However, the present study reveals the shift in the bacterial community of optimised *mahewu* produced with different inocula using a high-throughput microbiota determination approach. This research will serve as the foundation for standardised, high-quality production of white and yellow maize *mahewu*. Therefore, further studies are needed to identify organisms such as fungi and virus with the aim of developing novel healthy optimal *mahewu* products. Additionally, in order to establish the quality of optimal *mahewu* products, studies can be developed to determine the nutritional and phytochemical quality of these *mahewu* products. Additionally, future research should also focus on consumers acceptance of these products.

## Figures and Tables

**Table 1:** Statistical experimental design of independent variables for *mahewu* production using the Box–Behnken design of response surface methodology.

Experimental Runs	B_tm_ (min)	F_tp_ (°C)	F_tm_ (h)
1	15	25	16
2	15	25	72
3	10	25	44
4	20	25	44
5	10	35	72
6	15	35	44
7	15	35	44
8	20	35	16
9	15	35	44
10	15	35	44
11	10	35	16
12	20	35	72
13	15	35	44
14	10	45	44
15	15	45	16
16	20	45	44
17	15	45	72

Btm = Boiling time, Ftp = Fermentation temperature, Ftm = Fermentation time, °C = degree Celsius, h = hours and min = minutes.

**Table 2:** pH, TTA and TSS results of the *mahewu* samples.

F_tp_ (°C)	F_tm_ (h)	B_tm_ (min)	pH	TTA (%)	TSS (°Brix)
**White Maize**					
25	16	15	3.98 ^j^ ± 0.01	0.25 ^b^ ± 0	4.60 ^abc^ ± 0.17
25	72	15	3.50 ^a^ ± 0.01	0.50 ^e^ ± 0	5.03 ^e^ ± 0.15
25	44	10	3.56 ^b^ ± 0.01	0.50 ^e^ ± 0	4.60 ^abc^ ± 0
25	44	20	3.59 ^cd^ ± 0.01	0.50 ^e^ ± 0	4.43 ^a^ ± 0.29
35	16	10	3.98 ^j^ ± 0	0.25 ^b^ ± 0	4.60 ^abc^ ± 0.10
35	72	10	3.73 ^g^ ± 0.01	0.48 ^de^ ± 0.03	4.57 ^ab^ ± 0.15
35	16	20	4.03 ^k^ ± 0	0.20 ^a^ ± 0	5.90 ^g^ ± 0.10
35	72	20	3.71 ^f^ ± 0.01	0.48 ^de^ ± 0.03	4.97 ^de^ ± 0.06
35	44	15	3.76 ^h^ ± 0.02	0.48 ^de^ ± 0.03	5.43 ^f^ ± 0.06
35	44	15	3.62 ^e^ ± 0.01	0.48 ^de^ ± 0.03	4.73 ^bd^ ± 0.12
35	44	15	3.62 ^e^ ± 0.01	0.50 ^e^ ± 0	4.63 ^ab^ ± 0.15
35	44	15	3.60 ^d^ ± 0.01	0.47 ^d^ ± 0.03	4.93 ^de^ ± 0.06
35	44	15	3.57 ^c^ ± 0.01	0.47 ^d^ ± 0.03	4.83 ^de^ ± 0.06
45	16	15	4.20 ^n^ ± 0.01	0.20 ^a^ ± 0	4.83 ^de^ ± 0.06
45	72	15	3.90 ^i^ ± 0.01	0.40 ^c^ ± 0	5.30 ^f^ ± 0.20
45	44	10	4.08 ^m^ ± 0.01	0.20 ^a^ ± 0	4.60 ^abc^ ± 0
45	44	20	4.07 ^l^ ± 0.02	0.20 ^a^ ± 0	5.40 ^f^ ± 0.10
**Yellow Maize**					
25	16	15	4.01 ^j^ ± 0.01	0.25 ^b^ ± 0	4.43 ^ab^ ± 0.31
25	72	15	3.48 ^a^ ± 0.01	0.50 ^d^ ± 0	5.00 ^def^ ± 0
25	44	10	3.51 ^b^ ± 0.01	0.48 ^d^ ± 0.03	4.60 ^bc^ ± 0
25	44	20	3.56 ^c^ ± 0.01	0.50 ^d^ ± 0	5.43 ^gh^ ± 0.15
35	72	10	3.92 ^i^ ± 0.01	0.40 ^c^ ± 0	4.77 ^bcde^ ± 0.06
35	44	15	3.69 ^g^ ± 0.01	0.48 ^d^ ± 0	4.73 ^bcd^ ± 0.15
35	44	15	3.92 ^i^ ± 0.01	0.40 ^c^ ± 0	5.20 ^fgh^ ± 0.10
35	16	20	3.73 ^h^ ± 0.01	0.48 ^d^ ± 0.03	5.23 ^fgh^ ± 0.15
35	44	15	3.73 ^h^ ± 0.02	0.48 ^d^ ± 0.03	5.87 ^i^ ± 0.15
35	44	15	3.60 ^d^ ± 0	0.48 ^d^ ± 0.03	4.80 ^bcde^ ± 0.10
35	16	10	3.61 ^e^ ± 0.01	0.48 ^d^ ± 0.03	4.90 ^cdef^ ± 0.17
35	72	20	3.61 ^e^ ± 0.01	0.48 ^d^ ± 0.03	4.57 ^bc^ ± 0.12
35	44	15	3.63 ^f^ ± 0.01	0.48 ^d^ ± 0.03	4.10 ^a^ ± 0.36
45	44	10	4.87 ^l^ ± 0	0.20 ^a^ ± 0	5.47 ^bcd^ ± 0.05
45	16	15	5.28 ^n^ ± 0.01	0.20 ^a^ ± 0	4.63 ^efg^ ± 0.12
45	44	20	5.00 ^m^ ± 0.01	0.20 ^a^ ± 0	5.57 ^gh^ ± 0.15
45	72	15	4.34 ^k^ ± 0	0.20 ^a^ ± 0	5.13 ^hi^ ± 0.21

Ftp = Fermentation temperature, Ftm = Fermentation time and Btm = Boiling time. All data reported as mean ± SD (*n* = 3) for white and yellow maize. Alphabets in superscripts within a column show the significant difference among the pH, TTA = Titratable acidity and TSS = Total soluble solids. Significant at *p* ≤ 0.05.

**Table 3:** Coefficient of regression for the different mathematical model equations obtained for *mahewu* produced with white and yellow maize.

Coefficient	pH	TTA	TSS
**White Maize**			
β_0_	5.70414	−0.950000	3.56018
β_1_	−0.06275	0.043339	0.02193
β_2_	−0.02664 *	0.012366	0.00673
β_3_	−0.08255	0.063821	0.00871
β_11_	0.00113 *	−0.000725	−0.00111
β_22_	0.00019 *	−0.000089 *	0.00018
β_33_	0.00321	−0.002300 *	−0.00165
β_12_	0.00016	−0.000045	0.00004
β_13_	−0.00020	0.000000	0.00485
β_23_	−0.00013	0.000089	−0.00161
R^2^ (%)	94.31	90.37	60.58
**Yellow Maize**			
β_0_	7.47591	−1.12260	3.90414
β_1_	−0.30732 *	0.10172 *	−0.01424
β_2_	0.01592	0.00988	0.04825
β_3_	0.04174	−0.03239	−0.03851
β_11_	0.00542 *	−0.00146 *	0.00126
β_22_	0.00003	−0.00004	−0.00035
β_33_	−0.00074	0.00107	0.00781
β_12_	−0.00037	−0.00022	−0.00006
β_13_	0.00040	−0.00010	−0.00365
β_23_	−0.00077	0.00014	−0.00095
R^2^ (%)	90.45	89.80	31.99

β is the coefficient of various models and β_0_ represents the constant term, β_1_ and β_2_ are the linear effects of fermentation and cooking conditions. β_11_, β_22_ and β_33_ connote the quadratic effects and β_12_, β_13_ and β_23_ are the interactions. TTA = titratable acidity; TSS = total soluble solids. * Significant at *p* ≤ 0.05.

**Table 4:** Experimental values obtained for white and yellow maize *mahewu*.

WM	pH		TTA		TSS	
	BF	AF	BF	AF	BF	AF
MS	5.96 ^b^ ± 0.01	3.54 ^b^ ± 0.01	0.20 ^b^ ± 0	0.60 ^c^ ± 0	5.20 ^a^ ± 0.21	4.67 ^a^ ± 0.21
W	6.39 ^d^ ± 0.01	4.51 ^d^ ± 0.01	0.15 ^a^ ± 0.03	0.47 ^a^ ± 0.06	5.90 ^d^ ± 0.31	5.17 ^b^ ± 0.31
MM	5.90 ^a^ ± 0.02	3.41 ^a^ ± 0.02	0.20 ^b^ ± 0	0.68 ^d^ ± 0.03	5.61 ^c^ ± 0.15	5.03 ^ab^ ± 0.15
MWM	5.95 ^c^ ± 0.01	3.63 ^c^ ± 0.01	0.20 ^b^ ± 0	0.57 ^b^ ± 0.03	5.50 ^b^ ± 0.17	4.70 ^a^ ± 0.17
**YM**						
MS	5.97 ^b^ ± 0.02	3.47 ^b^ ± 0.02	0.20 ^b^ ± 0	0.60 ^bc^ ± 0	5.90 ^d^ ± 0.12	5.17 ^bc^ ± 0.15
W	6.41 ^c^ ± 0.01	4.65 ^c^ ± 0.01	0.15 ^a^ ± 0.03	0.38 ^a^ ± 0.03	5.50 ^b^ ± 0.12	4.93 ^a^ ± 0.12
MM	5.96 ^b^ ± 0.01	3.49 ^b^ ± 0.01	0.20 ^a^ ± 0	0.57 ^b^ ± 0.03	5.61 ^c^ ± 0.01	5.53 ^c^ ± 0.12
MWM	5.91 ^a^ ± 0.01	3.44 ^a^ ± 0.01	0.20 ^b^ ± 0	0.62 ^c^ ± 0.03	5.20 ^a^ ± 0.15	4.80 ^a^ ± 0.36

WM—White *mahewu*, MS—Malted Sorghum, W—Wheat, MM—Millet Malt, MWM—Malted White Maize, WMF—White Maize Flour, YM—Yellow *mahewu*, MYM—Malted Yellow Maize, YMF—Yellow Maize Flour, TTA—Titratable Acidity, TSS—Total Soluble Solid, BF—Before fermentation, AF—After fermentation. All data reported as mean ± SD (*n* = 3). Alphabets in superscripts within a column show significant difference among differently prepared *mahewu* samples. Significant at *p* ≤ 0.05.

## Data Availability

The data analysed and generated in this study is available from the corresponding author on reasonable request.

## References

[1] Siyuan S., Tong L., Liu R.H. (2018). Corn phytochemicals and their health benefits. Food Sci. Hum. Wellness.

[2] Palacios-Rojas N., McCulley L., Kaeppler M., Titcomb T.J., Gunaratna N.S., Lopez-Ridaura S. (2020). Mining maize diversity and improving its nutritional aspects within agro-food systems. Compr. Rev. Food Sci. Food Saf..

[3] Cuvas-Limon R.B., Nobre C., Cruz M., Rodriguez-Jasso R.M., Ruiz H.A., Loredo-Trevino A., Texeira J.A., Belmares R. (2020). Spontaneously fermented traditional beverage as a source of bioactive compounds: An overview. Cri. Rev. Food Sci. Nutr..

[4] Ogunremi O.R., Banwo K., Sanni A.L. (2017). Starter-culture to improve the quality of cereal-based fermented foods: Trends in selection and application. Curr. Opin. Food Sci..

[5] Gotcheva V., Pandiella S.S., Angelov A., Roshkova Z.G., Webb C. (2020). Microflora identification of the Bulgarian cereal-based fermented beverage boza. Process Biochem..

[6] Lappa I.K., Kachrimanidou V., Pateraki C., Koulougliotis D., Eriotou E., Kopsahelis N. (2020). Indigenous years: Emerging trends and challenges in winemaking. Curr. Opin. Food Sci..

[7] Kandylis P., Pissaridi K., Bekatorou A., Kenellaki M., Koutinas A.A. (2016). Dairy and non-diary probiotic beverages. Curr. Opin. Food Sci..

[8] Kumar R.S., Varman D.R., Kanmani P., Yuvaraj N., Paari K.A., Pattukumar V. (2010). Isolation, Characterization and Identification of a Potential Probiont from South Indian Fermented Foods (Kallappam, Koozh and Mor Kuzhambu) and its use as Biopreservative. Probiotics Antimicrob. Proteins.

[9] Setta M.C., Matemu A., Mbega E.R. (2020). Potential of Potential of probiotics from fermented cereal-based beverages in improving health of poor people in Africa. J. Food. Sci. Technol..

[10] Pswarayi F. (2019). Composition and Origin of the Fermentation Microbiota of *Mahewu*, a Zimbabwean Fermented Cereal Beverage. Appl. Environ. Microbiol..

[11] Awobusuyi T.D., Siwela M., Kolanisi U., Amonsou E.O. (2016). Provitamin A retention and sensory acceptability of *amahewu*, a non-alcoholic cereal-based beverage made with provitamin A-biofortified maize. J. Scie Food Agric..

[12] Fadahunsi I.F., Soremekun O.O. (2017). Production, nutritional and microbiological evaluation of *Mahewu* a South African traditional fermented porridge. J. Adv. Biol. Biotechnol..

[13] Adebo O.A., Njobeh P.B., Adebiyi J.A., Kayitesi E. (2018). Co-influence of fermentation time and temperature on physicochemical properties, bioactive components and microstructure of *ting* (a Southern African food) from whole grain sorghum. Food Biosci..

[14] Li H., Driesche S., Van D., Bunge F., Yang B., Vellekoop M.J. (2019). Optimization of on-chip bacterial culture conditions using the Box-Behnken design response surface methodology for faster drug susceptibility screening. Talanta.

[15] Simango C. (2000). Lactic acid fermentation of sour porridge and *mahewu*, a non-alcoholic fermented cereal beverage. JASSA.

[16] Gadaga T.H., Mutukumira A.N., Narvhus J.A., Feresu S.B. (1999). A review of traditional fermented foods and beverages of Zimbabwe. Int. J. Food Microbiol..

[17] Caro-quintero A., Konstantinidis K.T. (2012). Minireview Bacterial species may exist, metagenomics reveal. Environ. microbiol..

[18] Davies H. (2010). A role for “omics” technologies in food safety assessment. Food Control.

[19] Salmerón I., Thomas K., Pandiella S.S. (2015). Effect of potentially probiotic lactic acid bacteria on the physicochemical composition and acceptance of fermented cereal beverages. J. Funct. Foods.

[20] Xiong K., Chen Y., Shen S. (2019). Experimental optimization and mathematical modeling of supercritical carbon dioxide extraction of essential oil from Pogostemon cablin. Chin. J. Chem. Eng..

[21] Oyedeji A.B., Mellem J.J., Ijabadeniyi O.A., Mellem J.J., Ijabadeniyi O.A. (2018). Improvement of some quality attributes of soymilk through optimization of selected soybean sprouting parameters using response surface methodology. CyTA J. Food.

[22] Das S., Tamang J.P. (2021). Changes in microbial communities and their predictive functionalities during fermentation of toddy, an alcoholic beverage of India. Microbiol. Res..

[23] Mokoena M.P., Gqaleni N. (2016). Advantages of traditional lactic acid bacteria fermentation of food in Africa. Curr. Res. Technol. Educ. Top. Appl. Microbiol. Microb. Biotechnol..

[24] Mashau M.E., Jideani A.I.O., Maliwichi L.L. (2020). Evaluation of the shelf-life extension and sensory properties of *mahewu*–A non-alcoholic fermented beverage by adding Aloe vera (*Aloe barbadensis*) powder. Br. Food J..

[25] Muyanja C., Namugumya B.S. (2009). Traditional processing, microbiological phytochemical and sensory characteristics of *Kwete*, a Ugandan fermented maize based beverage. Afr. J. Food Agric. Nutr. Dev..

[26] Nanodoum M. (2010). Sorghum Beer: Production, Nutritional Value and Impact upon Human Health. Beer Health Dis. Prev..

[27] Ibrahim F.S., Babiker E.E., Yousif N.E., El Tinay A.H. (2005). Effect of fermentation on biochemical and sensory characteristics of sorghum flour supplemented with whey protein. Food Chem..

[28] Kutyauripo J., Parawira W., Tinofa S., Kudita I., Ndengu C. (2009). Investigation of shelf-life extension of sorghum beer (*Chibuku*) by removing the second conversion of malt. Int. J. Food Microbiol..

[29] Jay J.M., Loessner M.J., Golden D.A. (2005). Milk fermentation, fermented and nonfermented dairy products. Food Microbiol..

[30] Abriouel H., Omar N.B., López R.L., Martínez-Cañamero M., Keleke S., Gálvez A. (2006). Culture-independent analysis of the microbial composition of the African traditional fermented foods *poto poto* and *dégué* by using three different DNA extraction methods. Int. J. Food Microbiol..

[31] Song D.H., Chun B.H., Lee S., Son S.Y., Reddy C.K., Mun H.I. (2021). Comprehensive metabolite profiling and microbial communities of *doenjang* (Fermented soy paste) and *ganjang* (fermented soy sauce): A comparative study. Foods.

[32] Xie M., Wu J., An F., Yue X., Tao D., Wu R., Lee Y. (2019). An integrated metagenomic/metaproteomic investigation of microbiota in *dajiang-meju*, a traditional fermented soybean product in Northeast China. Food Res. Int..

[33] Schoustra S.E., Kasase C., Toarta C., Kassen R., Poulain A.J. (2013). Microbial Community Structure of Three Traditional Zambian Fermented Products: *Mabisi*, *Chibwantu* and *Munkoyo*. PLoS ONE.

[34] Teniola O.D., Odunfa S.A. (2002). Microbial assessment and quality evaluation of *ogi* during spoilage. World J. Microbiol. Biotechnol..

[35] Sohliya I., Joshi S.R., Bhagobaty R.K., Kumar R. (2009). *Tungrymbai*—A traditional fermented soybean food of the ethnic tribes of Meghalaya. Indian J. Tradit. Knowl..

[36] Kouamé A.K., Djéni T.N., N’Guessan F.K., Dje M.K. (2013). Postprocessing microflora of commercial *attieke* (a fermented cassava product) produced in the south of Côte d’Ivoire. Lett. Appl. Microbiol..

[37] del Carmen Portillo M., Mas A. (2016). Analysis of microbial diversity and dynamics during wine fermentation of Grenache grape variety by high-throughput barcoding sequencing. LWT Food Sci. Technol..

[38] Ni K., Minh T.T., Tu T.T.M., Tsuruta T., Pang H., Nishino N. (2017). Comparative microbiota assessment of wilted Italian ryegrass, whole crop corn and wilted alfalfa silage using denaturing gradient gel electrophoresis and next-generation sequencing. Appl. Microbiol. Biotechnol..

[39] Gaglio R., Cirlincione F., Di Miceli G., Franciosi E., Di Gerlando R., Francesca N. (2020). Microbial dynamics in durum wheat kernels during aging. Int. J. Food Microbiol..

[40] Huang Z., Shen Y., Huang X., Qiao M., He R.K., Song L. (2021). Microbial diversity of representative traditional fermented sausages in different regions of China. J. Appl. Microbiol..

[41] Nazari M., Mooraki N., Sedaghati M. (2021). Chemical and microbial properties of a fermented fish sauce in the presence of *Lactobacillus plantarum* and *Paenibacillus polymyxa*. Iran J. Fish Sci..

[42] Zhou J., Luo J., Yang S., Xiao Q., Wang X., Zhou Z. (2021). Different responses of microbiota across intestinal tract to *Enterococcus faecium* HDRsEf1 and their correlation with inflammation in weaned piglets. Microorganisms.

[43] Tao S., Bai Y., Zhou X., Zhao J., Yang H., Zhang S. (2019). In Vitro Fermentation Characteristics for Different Ratios of Soluble to Insoluble Dietary Fiber by Fresh Fecal Microbiota from Growing Pigs. ACS Omega.

[44] Tamang J.P., Shin D.H., Jung S.J., Chae S.W. (2016). Functional properties of microorganisms in fermented foods. Front. Microbiol..

[45] Kun-Young P., Ji-Kang J., Young-Eun L., James W.D. (2014). Health Benefits of Kimchi (Korean fermented vegetables) as a probiotic food. J. Med. Food.

[46] Dharaneedharan S., Heo M.S. (2016). Korean Traditional Fermented Foods—A Potential Resource of Beneficial Microorganisms and Their Applications. J. Life Sci..

[47] Ashaolu T.J. (2019). A review on selection of fermentative microorganisms for functional foods and beverages: The production and future perspectives. Int. J. Food Sci. Technol..

[48] Sullivanh T.F.O., Mahonyi A.O., Fitzgeraldw G.F., Sinderen D.V. (1999). A Comparative Study of Malthouse and Brewhouse Microflora. J. Inst Brew..

[49] Justé A., Crauwels S., Willems K., Cooman L.D., Lievens B., Aerts G. (2013). Assessing the xylanolytic bacterial diversity during the malting process. Food Microbiol..

[50] Waters D.M., Mauch A., Coffey A., Arendt E.K., Waters D.M., Mauch A. (2015). Lactic Acid Bacteria as a Cell Factory for the Delivery of Functional Biomolecules and Ingredients in Cereal-Based Beverages: A review. Crit. Rev. Food. Sci. Nutr..

[51] Mohammed S.S.D., Orukotan A.A., Musa J. (2017). Effect of fermentation and malting on some cereal weaning foods enriched with African locust beans. J. Appl. Sci. Environ. Manag..

[52] Krasauskas A. (2017). Fungi in malting barley grain and malt production. Biologija.

[53] Laureys D., Britton S.J., De Clippeleer J. (2020). Kombucha tea fermentation: A review. J. Am. Soc. Brew. Chem..

[54] Justé A., Malfliet S., Lenaerts M., De Cooman L., Aerts G., Willems K.A. (2011). Microflora during malting of barley: Overview and impact on malt quality. Brew. Sci..

[55] Hejazi S.N., Orsat V., Azadi B., Kubow S. (2016). Improvement of the in vitro protein digestibility of amaranth grain through optimization of the malting process. J. Cereal Sci..

